# Marked Tachycardia, complete right bundle branch block, and lactic acidosis following ingestion of Cannabidiol (CBD) gummies: A case report

**DOI:** 10.1016/j.toxrep.2025.102040

**Published:** 2025-04-26

**Authors:** Masatoshi Inoue, Miya Hiramatsu, Naoki Kamegai, Junichiro Yamamoto

**Affiliations:** a Department of Nephrology. Asahi University Hospital, 3-23 Hashimoto-cho, Gifu 500-8856, Japan; b Department of Nephrology. Nagoya University Graduate School of Medicine, Japan

**Keywords:** Cannabidiol, Cannabis, Complete Right Bundle Branch Block, Lactic Acidosis

## Abstract

The increasing availability of cannabidiol (CBD) products worldwide has raised concerns about their safety profile; although CBD is generally considered safe, adverse effects have been reported, particularly from overdose. This report describes a rare case of a 26-year-old male who ingested a small amount of CBD gummies (40 mg) and developed marked tachycardia, complete right bundle branch block (CRBBB), and lactic acidosis. The patient presented with nausea and vomiting, heart rate increased from a normal 80 bpm to 165 bpm, 12-lead ECG confirmed CRBBB, and laboratory tests showed elevated lactate levels indicative of type B lactic acidosis without shock or ischemia. Symptoms resolved with supportive care, including intravenous fluids, and the patient's cardiac conduction returned to normal within a week. This case highlights the potential for serious cardiovascular and metabolic complications from CBD gummy ingestion in Japan. These findings underscore the need for awareness among healthcare professionals of the potential side effects of CBD gummies, even in small doses. Close monitoring and supportive care are essential to manage these cases, and further research is needed to clarify the mechanisms of these effects and ensure the safety of CBD products.

## Highlights

• A healthy 26-year-old man developed tachycardia, CRBBB, and lactic acidosis after 40 mg CBD.
• ECG and metabolic changes resolved with supportive care alone, without specific treatment.
• Even low-dose CBD edibles may cause serious effects due to contamination or individual factors.
• Clinicians should monitor for CBD toxicity in patients with arrhythmia or lactic acidosis.

## Graphical Abstract

**Figure:**
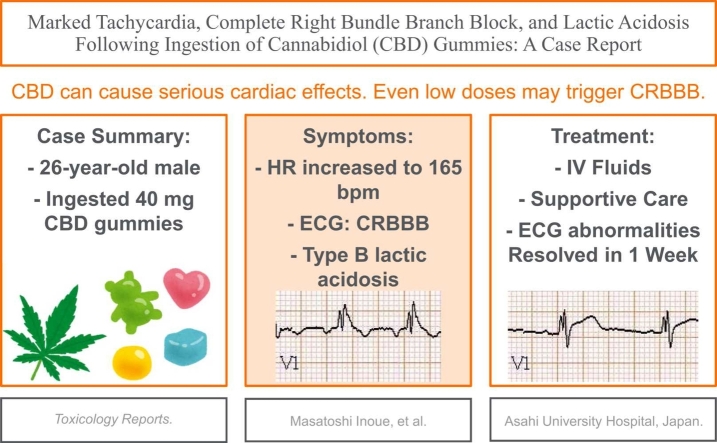

## 1 Introduction

The cannabis plant is rich in phytochemicals, with over 545 recognised substances and more than 140 cannabinoids [1]. The main components of Cannabis are tetrahydrocannabinol (THC) and Cannabidiol (CBD), and it is believed that the psychoactive effects are mainly caused by THC and not CBD, which is why CBD alone is legal in many countries. Clinically manufactured and quality-assured medical CBD formulations have been reported to be safe at relatively high doses in adults [2]. It has garnered interest within the medical community due to evidence suggesting its efficacy in the management of Dravet Syndrome and Lennox-Gastaut Syndrome. In recent years, it has been offered in a wide range of concentrations in various forms such as baked goods, gummies, and beverages (kombucha, beer, tea, etc.) called CBD edibles. However, CBD edibles are absorbed more slowly than inhalation, which presents the potential for an overdose. We report here a case of a 26-year-old man with no specific medical history who ingested 40 mg of CBD edible product for the first time and was admitted to the intensive care unit with tachycardia, complete right bundle branch block (CRBBB), vomiting and lactic acidosis. To our knowledge, there are only two cases of intoxication by CBD edible in the world. We discuss CBD edible intoxication, including its similarities and differences with cannabis intoxication.

## 2 Case presentation

**Fig. 1:**
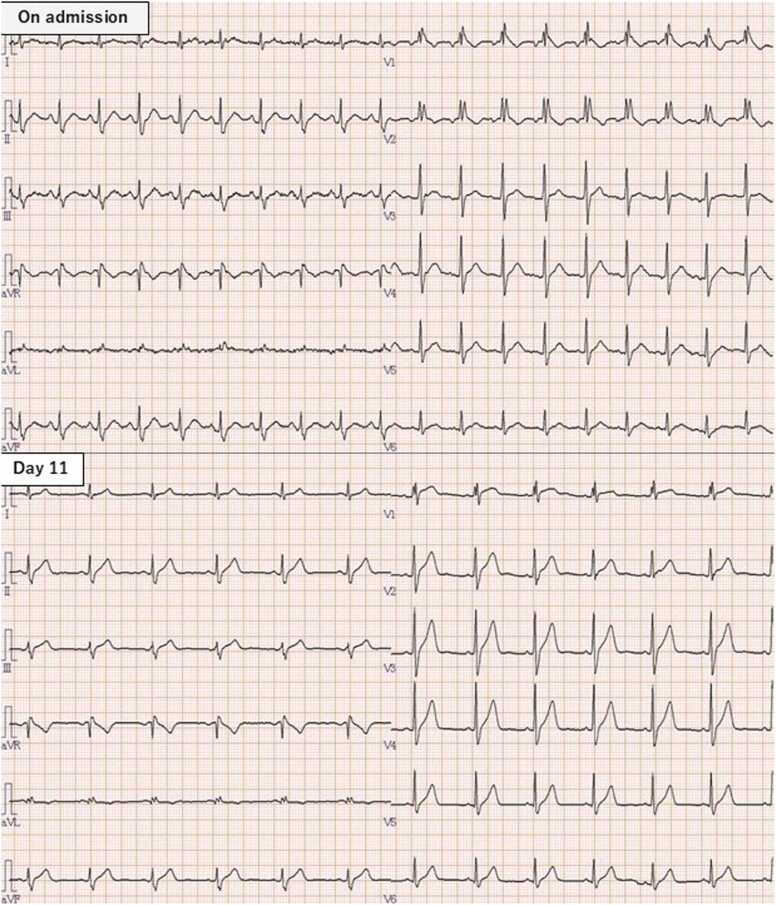
ECG on admission and day11. The QRS complex duration was 0.122 ms, and V1 and V2 leads showed ‘rsR’ pattern and negative T-waves on admission. This change had disappeared on day11.

A 26-year-old man was brought to our emergency department by ambulance due to complaints of nausea and vomiting. Upon arrival, his vital signs were as follows: body temperature of 36.1°C, blood pressure of 121/85 mmHg, heart rate of 125 beats per minute, respiratory rate of 22 breaths per minute, and oxygen saturation of 100 % on room air. His pupils were 3 mm, equal bilaterally, and reactive to light. Neurological examination revealed no limb paralysis. The abdominal examination showed a flat, soft abdomen with no tenderness. In the blood test, elevated lactate, elevated creatine kinase, and elevated blood glucose were observed (Table 1). A computed tomography of the abdomen revealed no abnormalities in the gastrointestinal tract or other abdominal organs. A 12-lead electrocardiogram (ECG) demonstrated a CRBBB (Fig. 1). All rapid urine drug tests (amphetamine, methamphetamine, morphine, codeine, 6-acetylmorphine, cocaine, benzoylecgonine, phencyclidine, methylenedioxyamphetamine, methylenedioxymethamphetamine, methylenedioxyethylamphetamine, and THC carboxylic acid) were negative. Approximately 20 minutes after admission, the patient’s heart rate progressively increased to 165 beats per minute, accompanied by repeated episodes of vomiting. Following episodes of copious vomiting, his heart rate spontaneously decreased to approximately 120 beats per minute, and his symptoms began to resolve. He was admitted to the intensive care unit for close monitoring and received 2500 ml of intravenous Ringer’s solution per day. One and a half hours after presentation, the heart rate decreased to 80 bpm, and the symptoms resolved spontaneously (Table 2). The patient was discharged the following day in stable condition. At a one-week follow-up, the complete right bundle branch block had resolved, and no recurrence of symptoms was noted.

**Table 1:** Laboratory investigations.

**Variable**	**Reference Range**	**On admission**	**Hospital Days 2**
Hemogrobin (g/dL)	11.6–14.8	16.5	15.0
Hematocrit (%)	35.1–44.4	48.2	44.3
White blood cell count (per μL)	3000–8600	5080	6070
Neutrophil count (per μL)	1800–7500	2160	3870
Lymphocyte count (per μL)	1000–4800	2380	1500
Eosinophil count (per μL)	100–300	7.6	42.5
Platelet count (per μL)	158,000–348,000	270000	212,000
Creatinine (mg/dL)	0.46–0.79	0.86	0.87
Urea nitrogen (mg/dL)	8–22	20.1	11.5
Creatine kinase (U/L)	59–248	366	254
Aspartate aminotransferase (U/L)	13–30	21	16
Alanine aminotransferase (U/L)	10–42	15	15
Albmin (g/dL)	4–5	4.5	3.8
C-reactive protein (mg/dL)	0–0.3	0.19	0.16
Sodium (mmol/L)	138–146	140	143
Potassium (mmol/L)	3.6–4.9	3.5	3.6
Chloride (mmol/L)	99–109	9.6	105
Glucose (mg/dL)	73–109	171	98
Venous blood gas			
pH	7.35–7.45	7.455	7.375
pCO2 (mmHg)	32–48	37.9	52.1
HCO3 (mmol/L)	21–28	26.3	29.8
Lac (mmol/L)	0.5–1.6	5.9	0.89

**Table 2:** Vital signs.

Hospital day	Time	Heart rate (Beats/min)	Blood pressure (mmHg)	Respiratory rate (Breaths/min)
1	21:30	125	121/85	22
1	21:50	165		28
1	22:00	120	123/85	24
1	23:00	81	126/68	15
2	0:00	88	134/80	16
2	7:00	81	104/87	15
2	8:00	80	106/74	17

## 3 Discussion

There have been only two reported cases of CBD gummy intoxication (Table 3). The first case took 370 mg of CBD gummies and showed HR 45 beats/minute bradycardia, respiratory depression and mild lactic acidosis (2.4 mmol/L) [3]; The second patient ingested 1600 mg of CBD gummies and showed HR 165 beats/minute tachycardia, lactic acidosis (4.1 mmol/L), and mildly elevated blood glucose (decreased over time) [4]. In this case, tachycardia, lactic acidosis and increased blood glucose were observed, similar to the second case. The dosage of CBD varies widely, and individual sensitivity to CBD may also exhibit significant variability. According to one review, factors influencing cannabinoid action include route of exposure, duration, frequency, drug interactions, age, gender and genetic polymorphisms of cannabinoid receptors and metabolizing enzymes [1].

**Table 3:** Summary of cases.

Case	Age	Sex	Max HR	Min HR	Lactate (mmol/L)	Amount of intake(mg)	Symptoms	Treatment
1	57	Male	78	78	2.4	370	Bizarre behavior and vomiting	Supportive care
2	29	Male	165	70	4.1	1600	Vomiting and quarreling	Intubation
This case	26	Male	165	80	5.9	40	Vomiting and restlessness	Supportive care

In reviews of cannabis edible intoxication, tachycardia has been reported in 84 % of cases, consistent with the presentation in this case [5]. On the other hand, there have also been reports of sick sinus syndrome associated with cannabis use [6]. In this case, the patient had a CRBBB that had not been previously noted on medical checkup and had normalized after one week. These findings suggest that the effects of cannabis or cannabinoids on the cardiac conduction system may also include inhibitory actions. As described below, type B lactic acidosis was complicated, and the metabolic changes in the tissue caused by CBD may have had a reversible effect on the conduction system of the myocardium. Hypertension is also often observed in cannabis intoxication, and in the present case the blood pressure was also increased by about 30 mmHg above the base line blood pressure. The reasons for the hypertension are still unknown, and further research is needed on the cardiovascular effects of cannabis and cannabinoids.

Lactic acidosis is also observed in a small number of cases of cannabis edible intoxication [7]. There have also been reports in CBD, which are thought to be due to increased aerobic glycolysis due to adrenergic hyperactivity and decreased lactate metabolism due to suppression of oxidative phosphorylation, but this is not certain [4]. The majority of cases of lactic acidosis are classified as type A lactic acidosis. Type A lactic acidosis is caused by the excessive production of lactic acid in ischemic tissues as a byproduct of anaerobic glycolysis, which is the process by which ATP (adenosine triphosphate) is produced during oxygen deprivation. In contrast, Type B lactic acidosis arises in the context of normal overall tissue perfusion, which is associated with normal ATP production. In this case, there was no evident shock or organ ischemia, and the lactic acidosis was classified as Type B. It was postulated that CBD or other adulterants may have inhibited metabolism.

The treatment for cannabis intoxication included the administration of intravenous fluids in 20 % of cases, dilute/irrigate/wash in 11 %, and benzodiazepines in 11 %. Additionally, 0.70 % of patients were intubated and managed [5]. In the cases of CBD previously discussed, one-three of patients, including this case, required intubation. However, the primary treatment strategy was supportive care. Because cannabinoids, including CBD, are highly liposoluble, they are sequestered in adipose tissue and have moderate to high volumes of distribution, varying from 2.5 to 10 L/kg, assuming a 70 kg adult body weight [8]. In addition, CBD binds strongly to plasma proteins and circulating blood cells. CBD is mainly metabolized hepatically by isozymes CYP2C19 and CYP3A4, as well as CYP1A1, CYP1A2, CYP2C9, and CYP2D6 in the liver. Therefore, it is difficult to remove by hemodialysis, and supportive care is expected to be the primary treatment.

Cannabis plant is known as a bioaccumulator, meaning that CBD products, including contaminants in the soil, contain a variety of non-CBD substances. CBD products have been reported to contain various contaminants. Heavy metals were detected in 25.7 % of the products, residual solvents in 89.6 %, and pesticides in 12.9 % [9]. In addition, a separate study demonstrated for the first time the presence of the endocrine-disrupting chemical phthalate esters in 13–80 % of the products [10]. As this shows, unexpected symptoms may occur that are not due to CBD. In Europe and the United States, a CBD preparation called epidiolex® is available for Lennox-Gastaut syndrome and Dravet syndrome. It has not been reported to cause lactic acidosis or tachycardia, and the symptoms in this case may have been caused by some other contaminant rather than CBD. In addition, a key limitation of this study is the inability to definitively rule out the intake of other substances or confirm the exact composition of the CBD product, as neither product analysis nor blood concentration measurements could be performed due to limitations in hospital facilities and funding.

## 4 Conclusion

It would seem that CBD edible products are becoming increasingly popular worldwide. It is therefore important that physicians are aware of the potential side effects. As the two previous reports and this case suggest, CBD edible intoxication can be successfully treated with appropriate systemic management and supportive care. However, close monitoring for serious cardiovascular and other effects may be necessary. However, CBD edible intoxication has only been reported in three cases, including the present case, and it cannot be ruled out that CBD edible is the sole cause, since contamination and individual metabolism may also be involved. Further case accumulation and detailed analysis are expected in the future.

## Authorship contribution

**Guarantor:** Masatoshi Inoue. **Conception and design of study:** M. Inoue, M. Hiramatsu, N. Kamegai, J. Yamamoto. **Acquisition of data:** M. Inoue, M. Hiramatsu, N. Kamegai, J. Yamamoto. **Analysis and/or interpretation of data:** M. Inoue, M. Hiramatsu, N. Kamegai, J. Yamamoto. **Drafting the manuscript:** M. Inoue. **Revising the manuscript critically for important intellectual content:** M. Inoue, M. Hiramatsu, N. Kamegai, J. Yamamoto. **Approval of the version of the manuscript to be published:** M. Inoue, M. Hiramatsu, N. Kamegai, J. Yamamoto.

## CRediT authorship contribution statement

**Yamamoto Junichiro:** Supervision, Project administration, Methodology, Investigation, Conceptualization. **Kamegai Naoki:** Supervision, Investigation, Formal analysis, Data curation, Conceptualization. **Hiramatsu Miya:** Project administration, Investigation, Data curation, Conceptualization. **Inoue Masatoshi:** Writing – original draft, Visualization, Validation, Supervision, Software, Resources, Project administration, Methodology, Investigation, Formal analysis, Data curation, Conceptualization.

## Ethical approval

We explained to the patient in person with a written consent form and obtained consent in writing signed by the patient.

## Declaration of Competing Interest

The authors declare that they have no known competing financial interests or personal relationships that could have appeared to influence the work reported in this paper.

## Data Availability

Data will be made available on request.

## References

[1] Kitdumrongthum S., Trachootham D. (2023 Mar 20). An individuality of response to cannabinoids: challenges in safety and efficacy of cannabis products. Molecules.

[2] Chesney E., Oliver D., Green A., Sovi S., Wilson J., Englund A., Freeman T.P., McGuire P. (2020 Oct). Adverse effects of Cannabidiol: a systematic review and meta-analysis of randomized clinical trials. Neuropsychopharmacology.

[3] Bass J., Linz D.R. (2020 Apr 16). A case of toxicity from Cannabidiol gummy ingestion. Cureus.

[4] Sheldon M., Ammons M., Nugent K. (2024 Oct 27). Acute toxicity with lactic acidosis associated with cannabinoid gummies. Cureus.

[5] Cao D., Srisuma S., Bronstein A.C., Hoyte C.O. (2016 Nov). Characterization of edible marijuana product exposures reported to United States poison centers. Clin. Toxicol..

[6] Iqbal A.M., Mubarik A., Cheetirala V.G., Mohammed S.K., Muddassir S. (2019 Jun 22). Marijuana induced sick sinus syndrome: a case report. Am. J. Case Rep..

[7] Hopkins L., Wilson M. (2023). Marijuana-induced lactic acidosis. Chest Crit. Care.

[8] Martinez Naya N., Kelly J., Corna G., Golino M., Polizio A.H., Abbate A., Toldo S., Mezzaroma E. (2024 Jan 18). An overview of Cannabidiol as a multifunctional drug: pharmacokinetics and cellular effects. Molecules.

[9] Gidal B.E., Vandrey R., Wallin C., Callan S., Sutton A., Saurer T.B., Triemstra J.L. (2024 Mar 18). Product labeling accuracy and contamination analysis of commercially available Cannabidiol product samples. Front Pharmacol..

[10] Gardener H., Wallin C., Bowen J. (2022 Dec 10). Heavy metal and phthalate contamination and labeling integrity in a large sample of US commercially available Cannabidiol (CBD) products. Sci. Total Environ..

